# Intimate partner violence against women and child maltreatment in a Brazilian birth cohort study: co-occurrence and shared risk factors

**DOI:** 10.1136/bmjgh-2020-004306

**Published:** 2021-04-30

**Authors:** Romina Buffarini, Carolina V N Coll, Terrie Moffitt, Mariângela Freitas da Silveira, Fernando Barros, Joseph Murray

**Affiliations:** 1 Postgraduate Program in Epidemiology, Department of Social Medicine, Federal University of Pelotas, Pelotas, RS, Brazil; 2 Human Development and Violence Research Centre, Federal University of Pelotas, Pelotas, Brazil; 3 International Center for Equity in Health, Federal University of Pelotas, Pelotas, RS, Brazil; 4 Social, Genetic, and Developmental Psychiatry Centre, Institute of Psychiatry, King's College, London, United Kinkdom; 5 Department of Psychology and Neuroscience, Duke University, Durham, North Carolina, USA

**Keywords:** child health, epidemiology, public health, cohort study

## Abstract

**Background:**

Intimate partner violence (IPV) against women and child maltreatment (CM) are major public health problems and human rights issues and may have shared causes. However, their overlap is understudied. We investigated the prevalence of IPV and CM, their co-occurrence in households and possible shared risk factors, in the general population of a Brazilian urban setting.

**Methods:**

Prospective population-based birth cohort, including over 3500 mother–child dyads with maternal reports on both IPV and CM when children were 4 years old. Eleven neighbourhood, family and parental risk factors were measured between birth and age 4 years. Bivariate and multivariate Poisson regression models with robust variance were used to test which potential risk factors were associated with IPV, CM and their co-occurrence.

**Results:**

The prevalence of any IPV and CM were 22.8% and 10.9%, respectively; the co-occurrence of both types of violence was 5%. Multivariate analyses showed that the overlap of IPV and CM was strongly associated with neighbourhood violence, absence of the child’s biological father, paternal antisocial behaviour in general and a mother–partner relationship characterised by high levels of criticism, maternal depression and younger maternal age. A concentration of many risk factors among 10% of the population was associated with a sixfold increase in risk for overlapping IPV and CM compared with households with no risk factors.

**Conclusion:**

IPV and CM share important risk factors in the family and neighbourhood environments and are particularly common in households with multiple social disadvantages and family difficulties. Integrated preventive interventions are needed.

**Key questions**

**What is already known?**

- Intimate partner violence (IPV) child maltreatment (CM) are two major public health issues and often occur together inside the same households.
- Evidence on IPV and CM show they are likely to share many risk factors, however, most studies have addressed the two issues separately.
- Studies comparing risk factors for IPV and CM or their overlap are extremely scarce, and no studies assessing the overlap among young children were found.

**What are the new findings?**

- The co-occurrence of IPV and CM within the same household was associated with neighbourhood violence, absence of the childs biological father, father antisocial behaviour, a poor mother–partner relationship, maternal depression and maternal young age.
- The concentration of multiple risk factors in the same household was associated with very high risk for IPV, CM and the co-occurrence of both types of violence.

**What do the new findings imply?**

- This study highlights the need to address violence against women and children as linked phenomena, particularly prevalent in the context of concentrated disadvantage.
- Researchers and policy-makers should aim for greater coordination of prevention programmes tackling IPV and CM.

## Introduction

Violence against women (VAW) and violence against children are two major public health issues.^1 2^ Worldwide, an estimated one in two children each year suffer some form of violence—such as bullying, violent discipline, emotional abuse, physical abuse, sexual abuse, neglect, fighting or community violence,^3^ and about 30% of women have ever experienced physical partner abuse or sexual violence,^4^ although prevalence can vary substantially by country and survey methodology.^3 5^ Physical and/or sexual intimate partner violence (IPV) is estimated to affect about 5%–45% of women each year in different low-income and middle-income countries (LMICs).^6^ The UN Sustainable Development Goals call for increased efforts to eliminate VAW and children. Despite the connections between children’s and women’s experiences of violence, most studies have addressed the two issues independently and overlooked how they often co-occur at home.^7^ Possible shared risks for IPV and child maltreatment (CM) suggest the potential for integrated interventions especially in contexts with high levels of both types of violence.^1 8–10^

There is significant overlap between IPV and CM in households,^11–18^ and increasing evidence suggests that this has compounding, intergenerational negative effects, both for women and children.^19 20^ Separate literatures on IPV and CM show they are likely to share many risk factors, including social norms condoning violence, household poverty and stress, marital conflict and parental use of alcohol and drugs.^1^ However, direct tests comparing risk factors for IPV and CM in the same study—in the same households—are extremely scarce. In a 1985 telephone survey in the USA, 8% of adults reported that there were both IPV and CM in the home.^21^ Participants reporting only IPV or only CM had many similar characteristics, but co-occurrence was particularly likely in the context of non-violent marital conflict and parental drug use. Two school surveys of Ugandan adolescents^20 22^ found substantial overlap between IPV and CM (present for about one quarter to one-third of the adolescents), and one survey^22^ showed this co-occurrence was associated with lower caregiver education, less emotional attachment in the partner relationship and higher caregiver mental distress and alcohol use. In short, there are very few recent representative studies of the overlap between IPV and CM and possible shared risk factors.

Notably, we found no previous study comparing risk factors for IPV and CM, or their overlap, for young children. Early childhood is a critical period of development, when nurturing care lays essential foundations for healthy outcomes through the life course and exposure to IPV and CM may fundamentally alter children’s biology and psychosocial trajectory.^23^ New studies of IPV, CM, their overlap and determinants are needed, especially in LMICs where household deprivation and violence is so prevalent and especially for young children whose development may be critically affected by exposure to violence.

In Brazil, both IPV and CM are very prevalent and CM severity is unusually high,^24–27^ making it a particularly important context to investigate both types of violence and their co-occurrence. However, only one study to date has investigated the links between IPV and CM in Brazil, in a specific population of 205 women and children (aged 0–18) attending health services in Rio de Janeiro.^28^ Our aim in the current study was to identify the extent of overlap between IPV and CM in a large, population-based birth cohort in southern Brazil and identify independent and shared risk factors for each type of violence.

## Methods

We conducted a birth cohort study in the city of Pelotas in southern Brazil, with around 340 000 inhabitants. In this 2015 Pelotas (Brazil) Birth Cohort Study, eligible children included all hospital-delivered children who were live-born in Pelotas between 1 January and 31 December 2015 and whose mother lived in the urban area of the city. Around 99% of children born in Pelotas are delivered in hospitals. From the 4333 eligible live births, 4275 were assessed at delivery, equivalent to a response rate of 98.7%. All these children and their mothers were invited to participate in follow-up assessments at 3, 12, 24 and 48 months, with a 95.4% response rate at the latest assessment at age 48 months. Information used in the current analyses was obtained from mothers (biological or social; non-maternal caregivers were excluded from analyses) using standardised questionnaires. Further information about the 2015 Pelotas Birth Cohort Study is available elsewhere.^29^

### Patient and public involvement

The public was not involved in the design or conduct of our research. Public was involved in the disseminations of the research. Measurements were conducted in confidential interviews and psychological support available when positive responses were given. All participants were informed of the general results through a study newsletter suitable for a non-specialist audience.

### Measures of IPV and CM

IPV and CM were measured in confidential interviews with mothers at the 48-month follow-up in a research centre. Information on IPV was collected using the instrument of the Multi-country Study on Women’s Health and Violence Against Women of the WHO.^30^ This instrument asks about acts occurring (yes/no) in the previous 12 months by a current or former partner and has three domains: emotional (four items), physical (six items) and sexual (three items). Each domain is scored positively if at least one of its constituent items is scored ‘yes’, and a final indicator of any IPV is scored positively if any of the 13 items is scored ‘yes’.

CM was measured using the Portuguese version of the Juvenile Victimization Questionnaire, 2nd edition, Screener Sum Version, Caregiver Lifetime Form (JVQ-R2).^31^ This is a very widely used instrument internationally, with extensive studies showing that respondents provide reliable and valid data in the context of careful research procedures.^32^ Cross-cultural adaptation and validation in Brazilian population has been conducted previously.^33 34^ In the current study, participants were asked to complete the questionnaire in a private interview with a trained female interviewer at the research centre. Any positive answers indicating possible maltreatment in the child’s lifetime instigated an interview with on-site psychologists hired to provide brief counselling and report any cases of current risk of abuse to social services. All participants who were interviewed by psychologists were provided with information about appropriate community support services. Based on this questionnaire, the following types of lifetime maltreatment were measured using the Child Maltreatment Module of the JVQ-R2, which includes a single item on each of: physical abuse, emotional abuse, neglect and family abduction/custodial interference; sexual assault by a known adult was also measured using the item in the Sexual Victimisation Module. Each question asks about lifetime victimisation (yes/no). As well as analysing individual types of maltreatment, an indicator of any CM was defined as having ever experienced at least one type of the five types of victimisations listed above. We investigated the possibility of collecting official records of maltreatment, but the number of cases of officially registered maltreatment in the city was extremely small, suggesting such official data would result in nearly all true cases being coded as false negatives and that maternal reports, shown elsewhere to produce high positive response rates,^32^ were the best source of information in this study.

### Measures of possible risk factors

The following were considered as possible risk factors for IPV and CM and their overlap: neighbourhood violence, family income, maternal and paternal education and age, father presence/absence, father antisocial behaviour, mother–father relationship quality, maternal depression, alcohol use and illicit drug use. These possible risk factors were chosen a priori based on reviews of risk factors for IPV,^35^ CM^36–38^ and, in particular, reviews of probable risk factors for their co-occurrence.^1 39^

Neighbourhood violence was assessed at the 48-month follow-up using questions^40^ scored 0–3 (never to often) about the frequency of four violent acts in the participant’s neighbourhood in the last 6 months: fights with weapons, fights between gangs, robbery and sexual violence. The scores were summed and then split into three categories, indicating increasing levels of violence (0–2, 3–7 and 8–12).

Information on family income, parental schooling and age was collected during the perinatal assessment. Family income, collected as a continuous variable, was obtained by summing the monthly income of all household members and then dividing the total into quintiles. Parents’ schooling was measured in complete years of formal education and then categorised into four groups (0–4, 5–8, 9–11 and ≥12). Mother’s and father’s age in complete years was classified as <20, 20–33 and ≥35.

Mothers provided information on whether the child’s father was living with the child at the 48-month assessment and specified whether the biological, adoptive or social father lived at home. The final variable used in the current analyses had three categories: (1) no father at home, (2) biological father at home and (3) social father at home. Twenty-four children with adopted fathers were excluded from this analysis, given this small number would not permit separate analysis, and combining adoptive fathers who are carefully screened by adoption agencies, with other social fathers, would not be appropriate. The category ‘no father at home’ means that no father figure (either biological or social) is living at home, even though the mother may have a partner outside the home.

Father antisocial behaviour was evaluated using the Antisocial Personality Module of the Mini International Neuropsychiatric Interview (MINI).^41–43^ Usually the MINI is used to measure the respondent’s own psychiatric symptoms, but in this study, mothers were asked about the behaviour of the child’s father—either biological or social, whoever had most contact with the child. A previous study by Caspi *et al* showed that mothers’ reports about paternal antisocial behaviours were reliable and valid.^44^ For the current analyses, we created a total father antisocial behaviour score by summing five of the questions (yes/no), but excluding the sixth question on domestic violence, in order to test the extent to which fathers’ general antisocial behaviour was a risk factor for IPV and CM. Fathers scoring 1+ were categorised as antisocial.

Mother–partner relationship conflict was assessed at 3 months, using two Likert-type questions^45^ about partners’ criticism of each other, ranging from 1 to 10 (low to high criticism). Each question asked how critical the mother considered her partner of her and how critical she considered herself to be of her partner. We first categorised each individual score as reflecting low (1–3), medium (4–6) or high (7–10) criticism, and then combined the two into a relationship criticism score coded as follows: low criticism (low–low or low–medium), medium criticism (medium–medium or low–high) or high criticism (medium–high or high–high).

Maternal depression was defined as scoring 13 or more points on the Edinburgh Postnatal Depression Scale, applied at 3 months, and previously validated in the same population.^46^ Finally, maternal alcohol drinking and drug use was assessed at 48 months with the Alcohol, Smoking and Substance Involvement Screening Test.^47 48^ Drug use was characterised as use of any illicit substance in the 3 months prior to interview, and alcohol use was defined as daily drinking of any alcoholic beverage.

### Analyses

The first set of analyses examined the prevalence and co-occurrence of IPV and CM. For twins, one sibling was excluded (n=48) and for one case of triplets, two siblings were excluded (n=2), so as to not count IPV against one mother multiple times. In the first descriptive analysis of individual outcomes, participants with valid data on each separate outcome (CM, n=3723; IPV n=3533) were included. For subsequent analyses of co-occurrence, mother–child pairs with complete information (n=3533) on the two outcomes—IPV and CM—were included. Co-occurrence is first visually represented in a Venn diagram. The extent of observed co-occurrence was compared with the statistically expected probability of co-occurrence due to chance (assuming that the two forms of violence are independent of each other). The expected prevalence of co-occurrence was estimated by multiplying the proportion of women who have experienced IPV by the proportion of children who experienced maltreatment. To test the significance of the difference between observed and expected co-occurrence, we compared the 95% CIs of the observed and expected prevalence rates. The observed and expected co-occurrence rates were reported as statistically different when the lower limit of the observed co-occurrence prevalence 95% CI was above the upper limit of the 95% CI for the expected co-occurrence. The 95% CI was calculated using the variance for the expected co-occurrence, defined as follows: varIPV−CM=varIPV×varCM+varIPV×(ECM)2+varCMt×(EIPV)2, where E=estimate and Var=SE^2^(n).

In the second set of analyses, IPV and CM were considered in relation to possible risk factors. For these analyses, in order to consider whether IPV and CM share risk factors, three separate outcomes were examined: only IPV (no CM reported), only CM (no IPV reported) and co-occurrence of both types of violence. For each of the three outcome variables, the reference group is the rest of the sample (eg, for the only IPV outcome variable, the reference group includes those experiencing no violence, only CM and co-occurrence). Prevalence ratios and 95% CI for the crude and adjusted associations between risk factors and violence outcomes were estimated using Poisson regression with robust variance.^49^

In adjusted analyses, risk factors were considered in seven hierarchal levels. The first level included neighbourhood violence. Levels 2–4 included the following socioeconomic and demographic variables: maternal and paternal age (level 2), maternal and paternal education (level 3) and family income (level 4). In the fifth level, father’s antisocial behaviour was added, and in the sixth level, father cohabitation with the child. The seventh level included behavioural, relationship and health characteristics that could have bidirectional associations. Therefore, when analysing the seventh level, separate models were estimated for each risk factor, without adjusting for other variables in the seventh level: mother–partner relationship (model 7a), maternal depression (model 7b), maternal alcohol use (model 7c) and maternal drug use (model 7d). The rationale for this adjusted analysis, adjusting for variables by hierarchical order, was to avoid adjusting for variables likely to represent mediating mechanisms between each risk factor and outcome.^50^ Variables with p<0.20 were kept in the model as possible confounders for subsequent levels. For levels 2 and 3, relevant variables were entered simultaneously in the adjusted analyses. Correlations between the variables were low (online supplemental table 1) and did not cause multicollinearity problems in the models.

10.1136/bmjgh-2020-004306.supp1 Supplementary data

Finally, the proportion of participants with each outcome was examined according to a cumulative risk score. For these analyses, first, each risk factor was dichotomised to compare the category representing the highest risk to all other categories. With this coding of 1 or 0 for every risk factor, a cumulative risk score was then calculated by summing across risk factors, and outcomes were examined according to this cumulative risk score.

Analyses were carried out in STATA V.14 (StataCorp, College Station, USA).

## Results

Information on IPV and CM was collected for 3533 mothers and 3723 children, respectively, representing 82.6% and 87.1% of the original participants in the cohort recruited at birth. The mean age of children was 45.5 months (SD=2.6) and 50.6% were boys (N=1886). Table 1 shows rates of overall IPV and overall CM in the study, as well as their individual subtypes. Overall, 22.8% of mothers reported some form of IPV in the previous year. The most prevalent form of IPV was emotional violence (21.7%), followed by physical violence (7.5%) and sexual violence (1.6%). Regarding CM, 10.9% of children were reported to have experienced any form of maltreatment in the child’s lifetime. Children were most commonly exposed to emotional abuse (7.8%), followed by family abduction/custodial interference, neglect and physical abuse (each with a prevalence of about 2%) and sexual assault by a known adult (0.2%). Comparing each form of violence between girls and boys, the only difference observed was a slightly elevated rate of overall CM for boys (11.9% for boys vs 9.8% for girls; online supplemental table 2).

**Table 1:** Prevalence of intimate partner violence (IPV) against women and child maltreatment (CM) in the 2015 Pelotas (Brazil) Birth Cohort Study

	N	% (95% CI)
Any IPV against women	804	22.8 (21.4 to 24.2)
Emotional violence	766	21.7 (20.4 to 23.1)
Physical violence	262	7.5 (6.6 to 8.3)
Sexual violence	55	1.6 (1.2 to 2.0)
Any CM	405	10.9 (9.9 to 11.9)
Psychological/emotional abuse	290	7.8 (7.0 to 8.7)
Physical abuse	62	1.7 (1.3 to 2.1)
Neglect	64	1.7 (1.3 to 2.2)
Family abduction/custodial interference	74	2.0 (1.6 to 2.5)
Sexual assault by known adult	9	0.2 (0.1 to 0.5)

Total number of mothers with valid data for IPV: 3533.

Total number of children with valid data for maltreatment: 3723.

**Figure 1:**
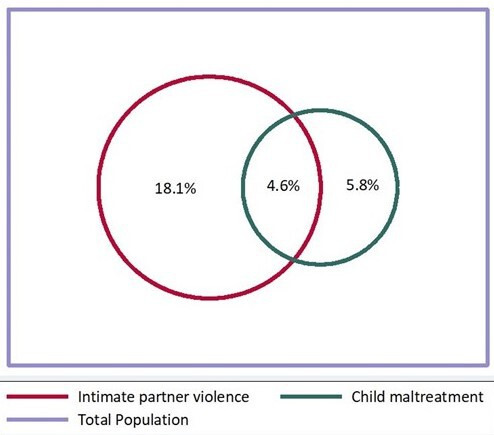
Venn diagram of intimate partner violence against women and child maltreatment in the 2015 Pelotas Birth Cohort (N=3533).

Figure 1 shows the extent to which IPV and CM overlapped within households or occurred in isolation. For 4.6% of the study population, there was an overlap—mothers reported both IPV and CM. This observed rate of co-occurrence is twice the rate compared with that which would be expected by chance (2.4%–95% CI 2.0 to 2.8), given the overall rates of IPV and CM in the study population (p<0.05 for the difference between observed and expected rates). This difference suggests that there are likely to be shared risk factors for IPV and CM.

Table 2 shows the rates of IPV only, CM only and their co-occurrence, according to family and parental characteristics and levels of neighbourhood violence. Five family and parent characteristics were strongly and significantly associated with all three violence outcomes: low family income, biological father not living with the child, father antisocial behaviour, poor mother–partner relationship and maternal depression. The following were associated with IPV and its co-occurrence with CM, but were not associated with CM on its own: neighbourhood violence, low paternal education and maternal use of illicit drugs. For CM on its own and in co-occurrence with IPV, there were strong associations with low maternal education and young age of the mother, but these risk factors did not associate with IPV alone.

**Table 2:** Intimate partner violence against women, child maltreatment and co-occurrence of both types of violence according to family and neighbourhood characteristics

	Total N	% only IPV	% only CM	% co-occurrence
Neighbourhood violence		P=0.005	P=0.394	P=0.005
0–2	2057	16.7	5.4	3.7
3–7	1196	19.3	6.6	5.6
8–12	279	24.0	6.1	7.2
Family income (quintiles)		P<0.001	P=0.078	P=0.035
Q1 (poorest)	659	24.0	5.9	6.2
Q2	741	18.9	7.8	5.5
Q3	716	18.9	5.9	4.5
Q4	721	15.8	5.4	3.6
Q5 (richest)	694	13.5	4.3	3.2
Maternal education (years)		P=0.154	P=0.001	P=0.001
0–4	298	19.8	10.7	7.1
5–8	893	19.8	5.6	6.5
9–11	1244	18.3	6.2	3.7
12 or more	1097	16.1	4.5	3.5
Maternal age (years)		P=0.839	P=0.135	P=0.032
<20	499	18.6	7.8	6.6
20–34	2524	17.9	5.5	4.5
≥35	509	18.9	5.9	3.1
Paternal education (years)		P=0.001	P=0.488	P=0.036
0–4	445	19.3	6.3	6.3
5–8	966	22.1	5.7	5.2
9–11	1089	17.5	6.0	3.6
≥12	854	14.9	4.6	3.5
Paternal age (years)		P=0.644	P=0.118	P=0.862
<20	194	20.6	8.8	4.6
20–34	2360	17.9	6.0	4.4
≥35	939	18.2	5.0	4.8
Father lives with child		P<0.001	P<0.001	P<0.001
No father at home	717	24.9	9.5	8.4
Social father at home	187	13.9	17.1	8.6
Biological father at home	2627	16.3	4.1	3.3
Father antisocial behaviour		P<0.001	P=0.003	P<0.001
Yes	683	35.0	8.4	11.6
No	2789	13.9	5.2	2.8
Mother–partner relationship		P<0.001	P=0.005	P<0.001
Low criticism	1975	14.1	4.0	2.8
Low-to-medium criticism	596	23.3	6.2	6.0
Medium-to-high criticism	426	29.8	7.3	6.8
Maternal depression		P<0.001	P=0.001	P<0.001
No	3113	17.0	5.3	3.7
Yes	361	27.4	10.0	11.6
Daily maternal use of alcohol		P=0.821	P=0.125	P=0.420
No	2805	19.2	5.6	5.2
Yes	34	17.7	11.8	8.8
Maternal use of illicit drugs		P<0.001	P=0.167	P=0.075
No	3409	17.7	5.8	4.5
Yes	123	30.9	8.9	8.1

Wald test p value.

Row percentages.

All variables have <5% missing data, except daily maternal use of alcohol (20%) and mother–partner relationship (17.5%).

Co-occurrence of any IPV and CM.

CM, child maltreatment; IPV, intimate partner violence against mother.

Table 3 shows associations between risk factors and the three violence outcomes, adjusting for other risk factors according to the hierarchical model. Nearly all risk factors were independently associated with violence against both women and children, even after controlling for confounders. The risk factors that were associated with all three violence outcomes (IPV only, CM only and their co-occurrence) were: biological father not living at home, father antisocial behaviour, poor maternal–partner relationship and maternal depression.

**Table 3:** Adjusted associations between potential risk factors and IPV, CM and the co-occurrence of both types of violence

Level	Variable	Only IPV	Only CM	Co-occurrence
		PR (95% CI)	PR (95% CI)	PR (95% CI)
1	Neighbourhood violence score	P=0.004	P=0.395	P=0.005
	0–2	Reference	Reference	Reference
	3–7	1.2 (1.0 to 1.3)	1.2 (0.9 to 1.6)	1.5 (1.1 to 2.1)
	8–12	1.4 (1.1 to 1.8)	1.1 (0.7 to 1.8)	1.9 (1.2 to 3.1)
2	Paternal age (years)	P=0.669	P=0.327	P=0.115
	<20	1.1 (0.8 to 1.6)	1.6 (0.8 to 3.0)	0.5 (0.2 to 1.1)
	20–34	1.0 (0.8 to 1.2)	1.4 (0.9 to 1.8)	0.7 (0.5 to 1.0)
	≥35	Reference	Reference	Reference
	Maternal age (years)	P=0.894	P=0.396	P=0.003
	<20	0.9 (0.7 to 1.3)	1.0 (0.6 to 1.8)	2.8 (1.5 to 5.4)
	20–34	0.9 (0.8 to 1.2)	0.8 (0.5 to 1.2)	1.5 (0.9 to 2.7)
	≥35	Reference	Reference	Reference
3	Maternal education (years)	P=0.932	P=0.005	P=0.19
	0–4	0.9 (0.7 to 1.2)	2.5 (1.3 to 4.3)	1.7 (0.9 to 3.4)
	5–8	1.0 (0.8 to 1.3)	1.3 (0.8 to 2.1)	1.4 (0.8 to 2.4)
	9–11	1.0 (0.8 to 1.2)	1.3 (0.8 to 1.9)	0.9 (0.6 to 1.5)
	12 or more	Reference	Reference	Reference
	Paternal education (years)	P=0.021	P=0.765	P=0.858
	0–4	1.3 (0.9 to 1.7)	0.9 (0.5 to 1.6)	1.1 (0.6 to 2.1)
	5–8	1.4 (1.1 to 1.8)	0.9 (0.6 to 1.5)	1.0 (0.6 to 1.8)
	9–11	1.1 (0.9 to 1.4)	1.1 (0.7 to 1.7)	0.9 (0.6 to 1.4)
	12 or more	Reference	Reference	Reference
4	Family income (quintiles)	P<0.001	P=0.055	P=0.915
	Q1 (poorest)	1.7 (1.3 to 2.2)	1.1 (0.6 to 2.0)	1.4 (0.7 to 2.7)
	Q2	1.3 (1.0 to 1.8)	1.8 (1.1 to 3.1)	1.3 (0.7 to 2.5)
	Q3	1.3 (1.0 to 1.7)	1.2 (0.7 to 2.2)	1.2 (0.6 to 2.2)
	Q4	1.1 (0.8 to 1.4)	1.2 (0.7 to 2.0)	1.1 (0.6 to 2.0)
	Q5 (richest)	Reference	Reference	Reference
5	Father antisocial behaviour	P<0.001	P=0.019	P<0.001
	Yes	2.4 (2.1 to 2.8)	1.5 (1.1 to 2.0)	3.9 (2.9 to 5.5)
	No	Reference	Reference	Reference
6	Father lives with child	P=0.048	P=<0.001	P=0.038
	No father at home	1.2 (1.0 to 1.4)	2.1 (1.4 to 2.9)	1.4 (1.0 to 2.2)
	Social father at home	0.7 (0.5 to 1.1)	3.5 (2.3 to 5.4)	2.0 (1.1 to 3.8)
	Biological father at home	Reference	Reference	Reference
7a	Mother–partner relationship	P<0.001	P<0.001	P=0.013
	Low criticism	Reference	Reference	Reference
	Low-to-medium criticism	1.5 (1.2 to 1.8)	1.5 (1.2 to 1.8)	1.7 (1.1 to 2.7)
	Medium-to-high criticism	1.9 (1.5 to 2.2)	1.9 (1.5 to 2.3)	1.8 (1.1 to 2.8)
7b	Maternal depression	P<0.001	P=0.007	P<0.001
	No	Reference	Reference	Reference
	Yes	1.5 (1.2 to 1.8)	1.7 (1.2 to 2.5)	2.4 (1.6 to 3.6)
7c	Maternal use of alcohol	P=0.109	P=0.109	P=0.671
	No	Reference	Reference	Reference
	Yes	0.4 (0.2 to 1.2)	2.1 (0.8 to 5.4)	1.3 (0.4 to 4.2)
7d	Maternal use of illicit drugs	P=0.065	P=0.521	P=0.339
	No	Reference	Reference	Reference
	Yes	1.3 (0.9 to 1.8)	1.2 (0.6 to 2.3)	1.4 (0.7 to 2.6)

Variables are adjusted for variables in all previous levels as well as other variables in the same level—except for variables in the seventh level, which are not adjusted for each other.

For each of the three outcome variables, the reference group is the rest of the sample (eg, for the only IPV outcome variable, the reference group includes those experiencing no violence, only CM and co-occurrence).

Co-occurrence of IPV and CM.

CM, child maltreatment; IPV, intimate partner violence against women; PR, prevalence ratio.

**Figure 2:**
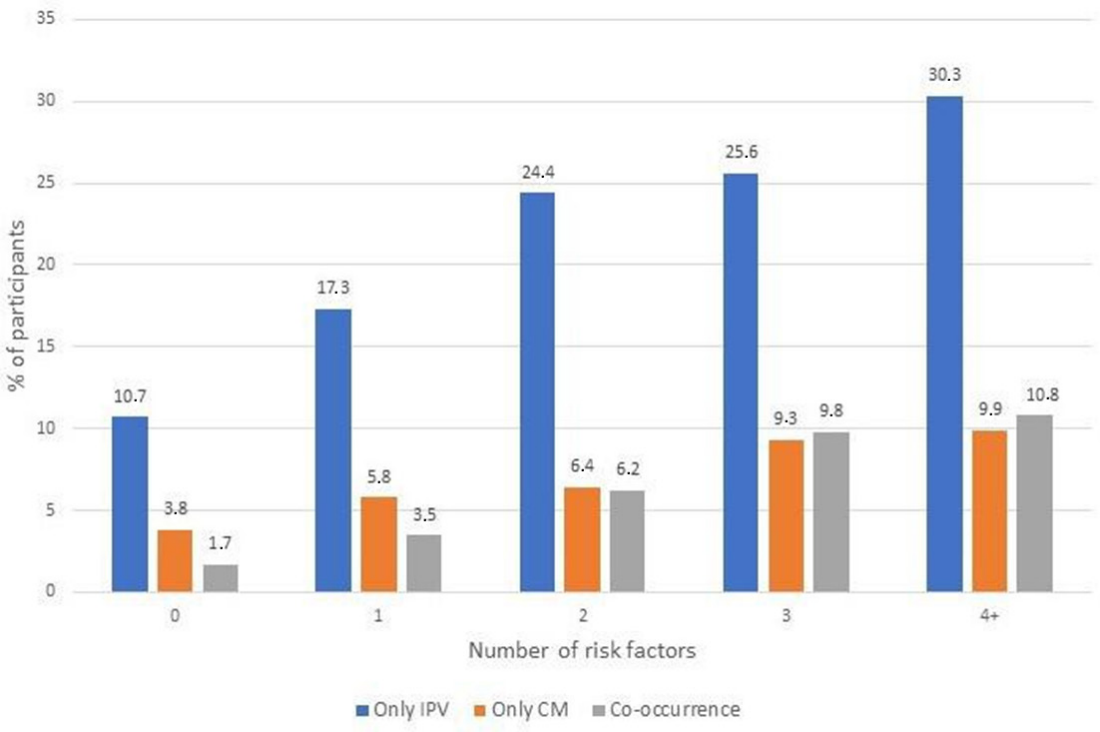
Prevalence of each of each type of violence outcome according to the number of risk factors in the household. CM, child maltreatment; IPV, intimate partner violence.

Summing women and children’s exposure across all the risk factors (based on the results in table 3, the ‘high risk’ categories chosen to calculate these ‘cumulative risk scores’ were as follows (high risk in brackets): neighbourhood violence (8–12); paternal age (<20); maternal age (<20); maternal education (0–4); paternal education (0–4); family income (lowest quintile); father antisocial (yes); father lives with child (no father at home); mother–partner relationship (medium–high criticism); maternal depression (yes); maternal alcohol (yes) and maternal drugs (yes)), cumulative risk scores in the population ranged from 0 to 8 (m=1.4; SD=1.4). Of all households, 34.9% (n=1303) had no risk factors; 26.5% (n 990) had one risk factor; 17.9% (n=669) had two risk factors; 10.8% (n=402) had three risk factors and 9.9% (n=366) had four or more risk factors. As figure 2 shows, there was a linear trend between the number of risk factors in the household and the likelihood of each violent outcome (IPV only, CM only as well as their co-occurrence). Among households with 4+ risk factors, one in two women reported either or both IPV and CM (30% of women experienced IPV only, 10% of children experienced CM only and 11% of households suffered both IPV and CM). Comparing households with 4+ risk factors to those with none, the increased risk for IPV only was 2.8 (95% CI 2.2 to 3.5, p<0.001); the increased risk for CM only was 2.6 (95% CI 1.7 to 4.0, p<0.001) and the risk of co-occurring IPV and CM was raised over sixfold (PR=6.4, 95% CI 3.8 to 10.6, p<0.001).

## Discussion

As far as we are aware, this is the first population-based study of young children to investigate risk factors for CM and maternal IPV and their overlap. Rates of IPV (22.8%) and CM (10.9%) in our study were high, although not as high as some other studies have reported,^3–6 51^ possibly due to heterogeneity in survey methodologies, participant age, settings, measures and definitions of violence. Some 5% of families experienced both forms of violence by the time children were 4 years old in our Brazilian setting. Risk factors contributing to the co-occurrence of IPV and CM included residing in a violent neighbourhood, absence of the biological father, father antisocial behaviour, poor mother–partner relationship, maternal depression and maternal young age. The concentration of multiple risk factors in the same household was associated with very high risk for all three violence outcomes: IPV only, CM only and the co-occurrence of both IPV and CM.

Our estimate that 5% of mother–child pairs experienced both IPV and CM is hard to compare with previous studies that have included measures of both, given the range of samples, types of violence examined (particularly for CM), reference periods and instruments used.^39 52^ However, uniformly studies have reported an increased risk for CM in the context of IPV.^11–14 16 18 20^ In a previous survey in Brazil, the co-occurrence of physical IPV and CM was 12.2% in a small sample of women and children attending health services in the city of Rio de Janeiro and was selected for a case–control study about domestic violence and premature birth.^28^ Across the global south, we could find only two previous studies of factors associated with the co-occurrence of IPV and CM, both in Uganda. One study of over 3000 Ugandan adolescents found that the combination of witnessing IPV and experiencing violence was associated with outcomes such as mental health problems, but was not associated with demographic characteristics of the household.^20^ The second study, including around 300 Ugandan adolescents, found that overlapping IPV and CM was associated with lower caregiver education and SES (socioeconomic status), lower partner attachment and higher mental distress and alcohol use among female caregivers and lower partner attachment as well as attitudes favouring violence against children among male caregivers.^22^ No studies of risk factors for IPV and CM among young children were found.

The current study empirically confirms, in a large population-based birth cohort, the theoretical proposition, derived principally from two parallel literatures, that IPV and CM share many risk factors. The shared risk factors in this Brazilian study point to the relevance of neighbourhood, family structure and household circumstances in increasing the risk for IPV, CM as well as the wider social and cultural influences implied by an ecological understanding of violence. Common aetiologies of IPV and CM involve stressful environments, problematic family relationships and male antisocial behaviour. Especially notable in the current study, elevated rates of both IPV and CM were found among families characterised by the absence of a biological father and by partner conflict, as has been reported previously in studies of IPV or CM alone.^19^ Social fathers have been found to perpetrate CM at higher rates than biological fathers,^53 54^ possibly because of lower attachment to non-genetically related children and related difficulties in developing roles as coparents.^55^ The presence of a stepfather was also associated with increased risk of child physical abuse in another Brazilian study.^56^ However, the benefits of living with two biological parents seems highly dependent on the quality of parental care and in particular levels of paternal antisocial behaviour.^57^ In the current study, paternal antisocial behaviour in general (ie, not necessarily directed at the mother or child) increased risk for both IPV and CM and their co-occurrence. Thus, difficult relationships with antisocial males, who are not necessarily children’s fathers, seem the most important risk factors for joint IPV and CM in this study. Association with violent men, while never the mother’s fault, has been understood to partly arise in the context of women’s own experiences of childhood violence, and maternal depression (also identified as a risk factor in our study) is considered one pathway to be addressed in the intergenerational transmission of CM.^58^

Critically, within some households there was a marked concentration of risk factors for family violence, with implications for intergenerational transmission.^59^ Exposure to multiple risk factors conferred increasing risk for both IPV and CM in a linear fashion. For the 10% of households with four or more risk factors, the likelihood of VAW and children was exceptionally high—half of mothers in such households reported IPV, CM or both, and co-occurring IPV and CM was six times more likely to occur for them than in households with no risk factors. In such vulnerable circumstances, individuals and families thus often experience multiple forms of violence that may be extremely difficult to address in isolation and without considerable support to face multiple family challenges. Although universal prevention programmes are important to alter social norms associated with violence and reduce risk factors across the population, intensive work with particularly vulnerable families, such as through multisystemic therapy with intersectoral support, is thus also critical to stem the intergenerational transmission of violence. In the current study, poorer, teenage and less educated mothers were at heightened risk for co-occurring IPV and CM and thus targeted interventions for younger disadvantaged women seem appropriate. Nurse home visiting programmes, such as the Nurse-Family partnership, have good evidence for reducing CM among young, disadvantaged women, and there is some evidence that this type of intervention may also reduce IPV.^60^

This study shows the need for both primary and secondary prevention strategies to reduce family violence, as well as treatment, especially in multiple-problem households where IPV and CM are most likely to co-occur. The exposure of young children to violence, both as victims of maltreatment and as witnesses to violence between adults, highlights the need for a life-course perspective, with longitudinal studies elucidating at which stages of development children are most exposed to violence and the optimum periods for intervention considering different possible determinants as children age.

This study has important strengths, including its urban population-based design, large sample, high follow-up rates, in-person interviews by trained research staff providing more reliable data on IPV and CM than official records and a wide range of possible risk factors in a longitudinal cohort. The findings should also be interpreted considering limitations of the study. First, results should not be generalised to rural populations. Second, as with all self-report studies of violence, there is likely to be under-reporting of violence in the current study. Third, there is possible reporter bias, as all information was provided by mothers, and this could contribute to overestimating associations. Reports on IPV or CM might also be biased by maternal mental health—and the association between violence and maternal depression in particular might be biased by using maternal reports only. Lastly, reverse causation is possible because many risk factors were measured simultaneously with family violence. For example, father absence could be a consequence of family violence, rather than a prospective risk factor. To minimise this bias, with IPV and CM as outcomes measured when children were age 4 years, wherever possible we selected measures of risk factors assessed previously in the cohort (eg, measures of partner relationship quality and maternal depression at 3 months postpartum); nonetheless, this does not rule out reverse causality. Bidirectional associations are likely to exist and this was considered in our hierarchical model, but we are unable to establish causal relationships in this study. Another limitation is that the current study did not identify the perpetrators of family violence.

## Conclusion

A false view that focusing on the problem of violence against children could undermine addressing VAW (and vice versa) has resulted in historically parallel but distinctive advocacy, research and response efforts. This study provides critical evidence that IPV and CM are prevalent and overlapping issues with several common risk factors and emphasises the need to address VAW and children as a joint phenomenon arising particularly in the context of concentrated disadvantage. Researchers and policy-makers should aim for greater coordination between IPV and CM prevention programmes to advance both fields in the best interests of women and children.

## Data Availability

Data are available upon request. Due to confidentiality restrictions related to the ethics approval for this study, no identifying information about participants may be released. Dataset without identification used during the current study is available from the corresponding author on reasonable request.
