# Supplementary material for: Intimate partner violence against women and child maltreatment in a Brazilian birth cohort study: co-occurrence and shared risk factors
Source: BMJ Glob Health. 2021 Apr 30;6(4):e004306. doi: 10.1136/bmjgh-2020-004306 (PMC8098765; doi:10.1136/bmjgh-2020-004306)
Supplement: Supplementary data [file bmjgh-2020-004306supp001.pdf]

Supplement Table 1. Tetrachoric correlation between possible risk factors for intimate partner violence and child maltreatment

duplicates list newid if ano==1	Neighbourhood violence	Family income	Maternal education	Maternal age	Paternal education	Paternal age	Father lives with child	Father antisocial behaviour	Mother-partner relationship	Maternal depression	Maternal alcohol use
Neighbourhood violence	1										
Family income	0.13	1									
Maternal education	0.24	0.49	1								
Maternal age	0.04	0.37	0.14	1							
Paternal education	0.26	0.40	0.59	0.17	1						
Paternal age	0.07	0.35	0.14	0.57	0.20	1					
Father lives with child	-0.03	0.21	0.09	0.25	0.12	0.20	1				
Father antisocial behaviour	0.19	0.09	0.08	0.08	0.11	0.15	0.56	1			
Mother-partner relationship	0.11	0.09	0.20	0.13	0.16	0.15	0.13	0.26	1		
Maternal depression	0.18	0.27	0.29	0.10	0.20	0.02	0.14	0.17	0.33	1	
Maternal alcohol use	-0.07	0.13	0.08	0.09	0.024	-0.70	0.18	0.05	0.05	0.23	1
Maternal illicit drug use	0.08	-0.01	0.14	0.22	0.02	0.20	0.35	0.28	0.17	0.25	0.03

Note. This table shows the tetrachoric correlations between the dichotomous risk factor variables used to create the cumulative risk score.

Supplement Table 2. Prevalence of intimate partner violence against women and child maltreatment according to child sex

	Boys		Girls		
	N	%	N	%	p*
Any intimate partner violence against women	402	22.5	402	23.0	0.779
Emotional Violence	384	21.4	405	21.8	0.838
Physical Violence	130	7.3	140	7.5	0.797
Sexual Violence	28	1.6	29	1.6	0.998
Any child maltreatment	225	11.9	180	9.8	0.036
Psychological/Emotional abuse	162	8.5	128	7.0	0.067
Physical abuse	37	2.0	25	1.4	0.160
Neglect	34	1.9	30	1.6	0.707
Family abduction/custodial interference	39	2.1	35	1.9	0.726
Sexual assault by known adult	4	0.2	5	0.3	0.751

*Fisher exact p-value for comparison of boys and girls

Total numbers of boys=1886; total number of girls=1844
